# Gastrointestinal cancer cells treatment with bevacizumab activates a VEGF autoregulatory mechanism involving telomerase catalytic subunit hTERT via PI3K-AKT, HIF-1α and VEGF receptors

**DOI:** 10.1371/journal.pone.0179202

**Published:** 2017-06-08

**Authors:** Nadine Mahfouz, Roula Tahtouh, Nada Alaaeddine, Joelle El Hajj, Riad Sarkis, Ray Hachem, Issam Raad, George Hilal

**Affiliations:** 1 Cancer and Metabolism Laboratory, Faculty of Medicine, Saint-Joseph University, Beirut, Lebanon; 2 Regenerative Medicine Laboratory, Faculty of Medicine, Saint-Joseph University, Beirut, Lebanon; 3 Faculty of Medicine, Saint-Joseph University and Hotel-Dieu de France, Surgery Department, Beirut, Lebanon; 4 Department of Infectious Diseases, the University of Texas M. D. Anderson Cancer Center, Houston, Texas, United States of America; Faculty of Medicine & Health Science, UNITED ARAB EMIRATES

## Abstract

**Background:**

Targeting angiogenesis has been considered a promising treatment of choice for a large number of malignancies, including gastrointestinal cancers. Bevacizumab is an anti-vascular endothelial growth factor (anti-VEGF) being used for this purpose. However, treatment efficacy is largely questioned. Telomerase activity, responsible for cancer cell immortality, is detected in 85–95% of human cancers and is considered a potential regulator of VEGF. The aim of our study was to investigate the interrelationship between VEGF and hTERT in gastrointestinal cancers and to explore cell response to a combined inhibition of telomerase and VEGF.

**Methods:**

AGS (gastric cancer), Caco-2 (colorectal cancer) and HepG2/C3A (hepatocellular carcinoma), were treated with telomerase inhibitors BIBR-1232 (10μM) and costunolide (10μM), with bevacizumab (Avastin® at 5 ng/ml or 100μg/ml) or with a combination of both types of inhibitors. VEGF and hTERT mRNA levels, and telomerase activity were detected by RT-PCR. VEGF levels were quantified by ELISA. Telomerase was knocked down using hTERT siRNA and hTERT was overexpressed in the telomerase negative cell line, Saos-2 (osteosarcoma), using constructs expressing either wild type hTERT (hTERT-WT) or dominant negative hTERT (hTERT-DN). Tube formation by HUVECs was assessed using ECMatrix™ (EMD Millipore).

**Results:**

Our results showed that telomerase regulates VEGF expression and secretion through its catalytic subunit hTERT in AGS, Caco2, and HepG2/C3A, independent of its catalytic activity. Interestingly, VEGF inhibition with bevacizumab (100μg/ml) increased hTERT expression 42.3% in AGS, 94.1% in Caco2, and 52.5% in HepG2/C3A, and increased telomerase activity 30-fold in AGS, 10.3-fold in Caco2 and 8-fold in HepG2/C3A. A further investigation showed that VEGF upregulates hTERT expression in a mechanism that implicates the PI3K/AKT/mTOR pathway and HIF-1α. Moreover, bevacizumab treatment increased VEGFR1 and VEGFR2 expression in cancer cells and human umbilical vein endothelial cells (HUVECs) through hTERT. Thus, the combination of bevacizumab with telomerase inhibitors decreased VEGF expression and secretion by cancer cells, inhibited VEGFR1 and VEGFR2 upregulation, and reduced tube formation by HUVECs.

**Conclusions:**

Taken together, our results suggest that bevacizumab treatment activates a VEGF autoregulatory mechanism involving hTERT and VEGF receptors and that an inhibition of this pathway could improve tumor cell response to anti-VEGF treatment.

## Introduction

Gastric, liver, and colorectal cancers are among the most common types of cancer and represent the major burden of cancer-related deaths worldwide [1–3]. Human telomerase is a ribonucleoprotein complex present in 85–95% of human cancers [4,5]. In addition to its pivotal role in ensuring telomere maintenance, a large number of studies have provided evidence for the non-telomeric functions of its catalytic subunit, hTERT. These functions include regulation of gene expression, cellular signaling [6–8], cell cycle regulation [9], inhibition of apoptosis [10], modulation of DNA damage response [11], cell metabolism [12], and mitochondrial function [13–16].

Vascular endothelial growth factor (VEGF) is known as the most potent and cell specific angiogenic factor [17,18]. VEGF induces angiogenesis through a mechanism involving activation of a signaling cascade in cells expressing the two major VEGF receptors (VEGFRs), VEGFR-1 and VEGFR-2 [17,19]. Binding of VEGF to VEGFR2 activates downstream survival and migration pathways involving PI3-kinase/AKT, focal adhesion kinase [20], and MAPK [21].

As the major mediator of tumor angiogenesis, VEGF and its receptors have been considered as possible targets to inhibit tumor angiogenesis and reduce tumor growth in several cancers. Bevacizumab (Avastin®) is a humanized VEGF monoclonal antibody that targets tumor angiogenesis. Bevacizumab was the first anti-angiogenic agent approved for anti-angiogenic therapy [22], and has shown benefit in several solid tumors [23,24]. However, bevacizumab treatment has been associated to a number of toxicities including hypertension, proteinuria, gastrointestinal perforations, and bleeding [25]. Moreover, several studies have reported that the survival benefits of bevacizumab treatment are modest and sometimes followed by relapses due to increased invasion and resistance mechanisms [26–28].

A correlation between telomerase and VEGF expression has been established by a number of studies [21,29,30]. These studies suggested the presence of an autoregulatory loop between VEGF and telomerase, while other studies detected the presence of VEGFR1 and/or VEGFR2 in cancer cells [31–34]. These studies have shown that VEGF also acts as an autocrine growth and survival factor for VEGF receptor-expressing tumor cells.

For a wider application, bevacizumab’s mode of action should be more established, and combinations with a better therapeutic index need to be developed. In this report, we studied the effect of bevacizumab treatment on telomerase expression and activity in gastrointestinal cancer cells and investigated whether a combined inhibition of VEGF and telomerase could reduce angiogenesis. We observed that VEGF inhibition with bevacizumab triggers an increase in hTERT, VEGFR1 and VEGFR2 expression and that a combined inhibition of VEGF and hTERT in both gastrointestinal cancer cells and endothelial cells could lead to a better anti-angiogenic therapeutic outcome.

## Materials and methods

### Cell lines and culture conditions

This research was conducted with ethical approval from the Saint Joseph University Ethical Committee and was carried out in accordance with the approved guidelines. Three telomerase-positive cell lines: Gastric adenocarcinoma (AGS), colorectal adenocarcinoma (Caco-2) and hepatocellular carcinoma (HepG2/C3A); and one telomerase-negative osteosarcoma cell line, Saos-2, were purchased from ATCC (American Type Cell Culture, Manassas, VA, USA). Cells were cultured in Dulbecco’s Modified Eagle Medium (Sigma-Aldrich, Germany) supplemented with 10% fetal bovine serum (FBS) and 1% Penicillin/Streptomycin and were incubated in a 5% CO_2_ humid incubator at 37°C. Human umbilical vein endothelial cells (HUVECs) were obtained from human umbilical cords of patients who delivered babies at Hotel Dieu de France Hospital, after obtaining a written consent form. The use of the umbilical cords for endothelial cells extraction was approved by the Saint Joseph University Ethics committee. HUVECs were isolated within 3 hours of collagenase digestion. Detached cells were washed in Phosphate Buffered Saline (PBS) and were cultured in endothelial cell growth media EGM Bulletkit™ medium (Lonza, Belgium) supplemented with bovine brain extract, ascorbic acid, hydrocortisone, human epidermal growth factor (hEGF), FBS and Gentamicin/Amphotericin-B. Flow cytometry analysis showed that the extracted HUVECs were positive for CD-31 and VEGFR2 (S1 Fig).

### Treatment with VEGF, Telomerase inhibitors and PI3K-AKT pathway inhibitors

Cells were treated with Bevacizumab (Avastin®), provided by Hoffmann-La Roche Ltd. (Switzerland), at 5 ng/ml and 100 μg/ml, telomerase inhibitors BIBR-1532 (10 μM) and costunolide (10 μM), PI3K inhibitor PI 828 (10μM), AKT inhibitor GSK 690693 (100 nM), mTOR inhibitor Rapamycin (200 nM), and HIF-1α inhibitor KC7F2 (40 μM) from Tocris (Tocris Bioscience, United Kingdom).

### Cell proliferation analysis

Cell proliferation was determined using a 2-(2-methoxy-4-nitrophenyl)-3-(4-nitrophenyl)-5-(2,4-disulfophenyl)-2H-tetrazolium, monosodium salt (WST-8) cell counting kit (Sigma-Aldrich, Germany). Briefly, 10^5^ cells were plated in each well of a 96-well plate in quadruplicate, allowed to grow for 48 hours, and then treated with different inhibitors for another 48 hours. Tetrazolium salt was then added to each well, and the formazan formation was assessed using an ELISA reader at 450 nm. Cell proliferation was also examined using trypan blue (Sigma-Aldrich, Germany).

### RNA extraction and reverse-transcription polymerase chain reaction (RT-PCR)

Total RNA was extracted using the NucleoSpin® RNA extraction kit (Macherey-Nagel, Germany), as recommended by the manufacturer. Total RNAs were reverse transcripted using the iScript cDNA Synthesis Kit (Bio-Rad, USA). Genes of interest were amplified using 5x FIREPol® Master Mix (Solis BioDyne, Estonia). Primer sequences for *hTERT*, *VEGF* and *GAPDH* amplification were: *hTERT* forward primer 5’-TGAACTTGCGGAAGACAGTGG-3’, *hTERT* reverse primer 5’-ATGCGTGAAACCTGTACGCCT-3’, *VEGF* forward primer 5’-GGAGGGCAGAATCATCACGAAG-3’, *VEGF* reverse primer 5’-CACACAGGATGGCTTGAAGATG-3’, *GAPDH* forward primer 5’-TGGGATGGACTGTGGTCATGAG-3’, *GAPDH* reverse primer 5’-ACTGGCGTCTTCACCACCATGG-3’. Reactions were initiated at 95°C for 3 min, followed by 94°C, 58°C (for *hTERT*), or 60°C (for *VEGF* and *GAPDH*) for 30 s, and 70°C for 30 s, 40 cycles for *hTERT*, or 35 cycles for *VEGF* and *GAPDH*, then followed by an elongation step at 72°C for 7 min. After amplification, PCR products were separated on 2% agarose gels and visualized by SYBR® Safe DNA Gel Stain (Invitrogen, Life Technologies, USA).

### siRNA analysis

The small interfering RNAs (siRNAs) against hTERT, non-silencing siRNA (control siRNA), and cell death siRNA were purchased from Qiagen (Qiagen Inc.). hTERT siRNA sequences were: 5’-GGAGCAAGUUGCAAAGCAUTT-3’ (sense) and 5’-AUGCUUUGCAACUUGCUCCAG-3’ (antisense). Briefly, 2 x 10^5^ cells were transfected in 6-well plates with 20 μM siRNAs using HiPerFect Transfection Reagent (Qiagen), according to the manufacturer’s fast-forward protocol for 24h, 48h, and 72h. Cell death siRNA were used as a positive control for transfection. Specific downregulation of hTERT was confirmed afterwards by RT-PCR.

### hTERT-WT and hTERT-DN expression constructs

Wild-type hTERT (*hTERT-WT)* and dominant-negative hTERT mutant (*hTERT-DN*) expression plasmids pBabe-neo-hTERT and pBabe-puro-DN-hTERT, respectively, were a gift from Bob Weinberg (Addgene plasmid #1774 and #1775) [35], while the empty vector pBabe-neo, was a gift from Hartmut Land & Jay Morgenstern & Bob Weinberg (Addgene plasmid # 1767) [36]. Plasmids were purified from the transformed E.coli DH5alpha bacteria with a GenElute Plasmid Miniprep Kit and then with a GenElute HP Plasmid Maxiprep kit (Sigma-Aldrich, USA). Cells were then transfected using the fast-forward protocol with Attractene Transfection Reagent (Qiagen Inc.) for 24h and 48h, according to the manufacturer’s instructions.

### VEGF-165 quantification by ELISA

The levels of VEGF secreted into the culture medium from AGS, Caco-2, HepG2/C3A, and Saos-2 cells were quantitatively measured using the Quantikine Human VEGF ELISA Kit (R&D Systems, Minneapolis, USA), according to manufacturer’s instructions. Briefly, 2x10^5^ cells were seeded in each well of a 6-well plate and treated at 80% confluence, as described in the figure legends. The supernatant was then collected and assayed. The optical density (O.D.) that is proportional to VEGF concentration was measured using an ELISA reader (Thermo Fisher Scientific, Inc.) at 450 nm.

### Assay of Telomerase activity

Telomerase activity was assayed using the Quantitative Telomerase Detection Kit (Allied Biotech, Inc.), according to manufacturer’s instructions. Briefly, 10^5^ cells were trypsinized and centrifuged at 2000 rpm for 5 min; then, cell pellets were washed with PBS and resuspended in 200 μl lysis buffer. Cell suspension was incubated on ice for 30 min and then centrifuged at 12000 g for 30 min at 4°C, and cell lysates were collected. One microliter of cell lysate was used for real-time PCR reaction. Heat inactivated samples were used as negative controls.

### Quantitative real-time PCR (qPCR)

To ascertain the effect of telomerase on the transcription rates of VEGFR1 and VEGFR2, quantitative real-time polymerase chain reaction (qRT-PCR) was performed. cDNA samples were amplified using the QuantiFast SYBR Green PCR Kit (Qiagen). The VEGFR1 primers used were VEGFR1-F (CAGGCCCAGTTTCTGCCATT) and VEGFR1-R (TTCCAGCTCAGCGTGGTCGTA), VEGFR2-F (CCAGCAAAAGCAGGGAGTCTGT) and VEGFR2-R (TGTCTGTGTCATCGGAGTGATATCC), GAPDH-F (5’ TCATCATCTCTGCCCCCTCT 3’), and GAPDH-R (5’ TCCGACGCCTGCTTCACCAC 3’), as internal controls. cDNA was amplified using a PCR program of 35 cycles, with denaturation at 95°C for 10 s, annealing at 62°C for 30 s, elongation at 72°C for 10 s and followed by a melting curve from 55°C to 95°C, using a Rotor-Gene Q PCR cycler (Qiagen, Germany).

### VEGFR2 quantification by ELISA

VEGFR2 was quantified using the Human VEGFR2/KDR Quantikine ELISA Kit (R&D Systems, Minneapolis, USA). According to the manufacturer’s instructions, assayed cells were washed with cold PBS, resuspended in the cell lysis buffer, incubated for 1 hour at room temperature and then centrifuged at 1000 x g for 15 mins. The supernatant was then assayed, and the enzymatic reaction was detected at 450 nm.

### Electrophoretic mobility shift assays (EMSA)

Single-stranded biotin-labeled oligonucleotides containing HIF-1α consensus DNA binding site were purchased from Bio-Rad (USA). Double-stranded oligonucleotides were prepared by annealing complementary oligonucleotides in a buffer containing 10 mM Tris (pH 8.0), 50 mM NaCl, and 1 mM EDTA. The sequences of the complementary pairs are as follows: TERT1, 5’- BIOTEG- GCGCTCCCCACGTGGCGGAGGG-3’, and HRE 1, 5’ -CCCTCCGCCACGTGGGGAGCGC-3’, TERT2, 5’- BIOTEG-CTGCTGCGCACGTGGGAAGCCC-3’, and HRE 2, 5’-GGGCTTCCCACGTGCGCAGCAG-3’. For the EMSA, labeled double-stranded oligonucleotides were incubated with nuclear cell extracts in a binding buffer containing 10 mM Tris (pH 8.0), 1mM EDTA, 50 mM NaCl, BSA (1.45 mg/ml), glycerol and poly d(I-C) (1 μg/μl).

### Flow cytometry analysis of PI3K, AKT, and mTOR protein levels

To determine the intracellular protein levels of PI3K, AKT, and mTOR following bevacizumab treatment, cells were washed twice with PBS, then fixed and permeabilized using the Cytofix/Cytoperm™ Kit (BD, USA) according to the manufacturer’s instructions. Briefly, cells were incubated with a fixation permeabilization solution for 20 minutes at 4°C. After incubation, cells were washed twice with Perm/Wash™ buffer, and then stained with the appropriate monoclonal antibodies against human PI3K, AKT, and mTOR (Abcam), for 30 minutes at 4°C in the dark. Finally, the cells were washed twice with Perm/Wash™ buffer and re-suspended in ranging buffer for the flow cytometry analysis.

### In vitro angiogenesis assay

According to the manufacturer’s instructions, 50 μl of growth factor-reduced extracellular matrix, ECMatrix™ (EMD Millipore, Darmstadt, Germany), was added to each well of a 96- well plate and gelation was undertaken at 37°C for 1 hour. Briefly, 5x10^3^ of treated HUVECs (5x10^3^) suspended in 100 μl EGM were added to the wells coated with Matrigel. Plates were incubated at 37°C in a humidified chamber of 5% CO2 for 4 hours to allow tube formation. Tube formation images were captured using an inverted microscope (Olympus, Tokyo, Japan), and tube structures were quantified by counting branch points in 3 random fields from each well. The average number of branch points was calculated, and each experiment was repeated in triplicate.

### Statistical analysis

All graphed data are presented as the mean ± SE from at least three experiments. Unpaired, two-tailed Student’s t-test was performed to determine significance. A p-value of ≤ 0.05 was considered significant. P-value ≤0.05 was considered significant; * represents p-value ≤0.05, ** as p-value ≤0.01.

## Results

### Telomerase inhibition decreases VEGF expression and secretion

**Fig 1:**
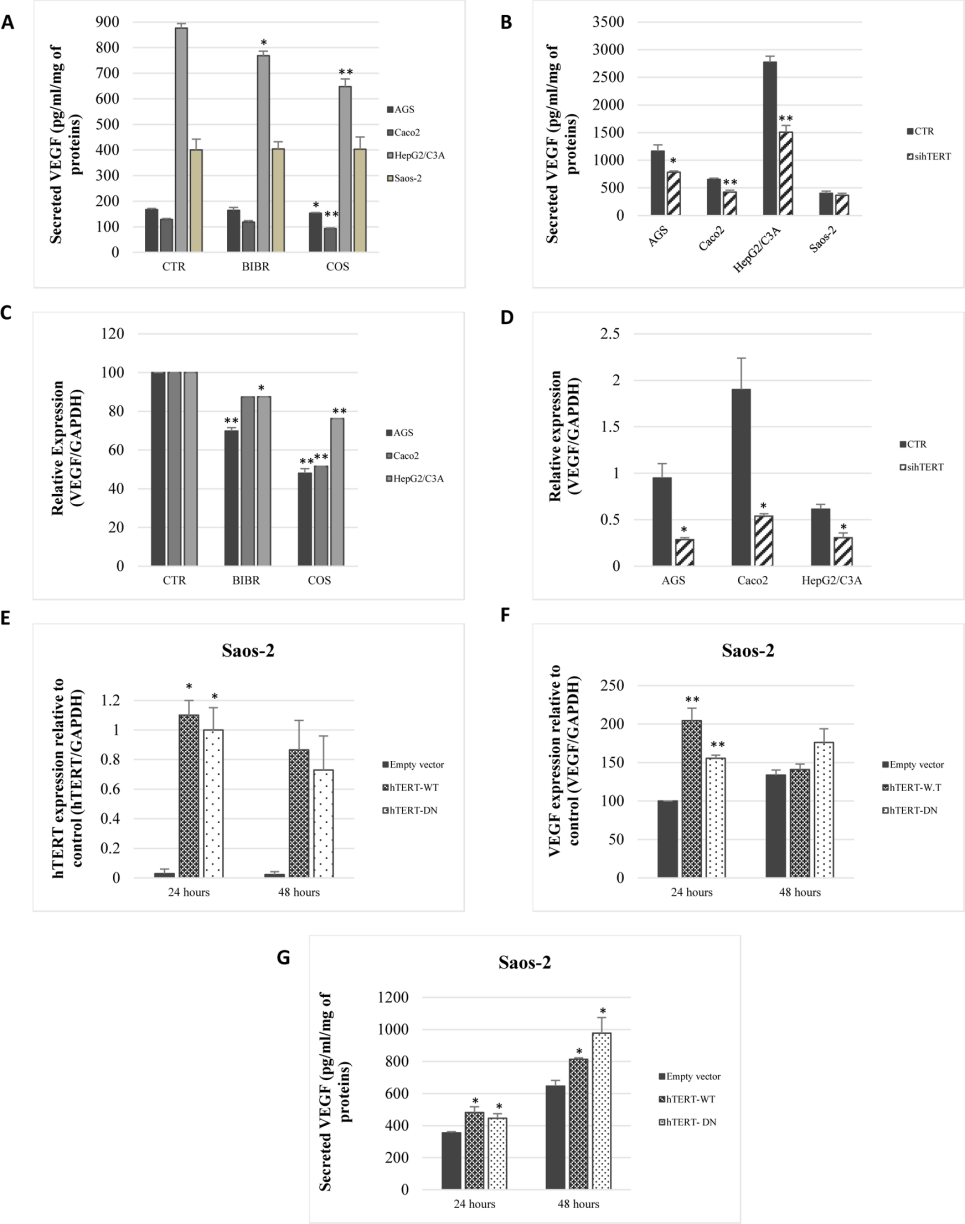
Telomerase regulates VEGF secretion and expression independently of telomerase activity. (A, B) VEGF secretion was assessed by ELISA following a 48-h treatment with telomerase inhibitors BIBR-1532 (10 *μ*M) and costunolide (10 *μ*M), and after 72 h of hTERT knockdown with siRNA. (C, D) Total RNAs were isolated from the cells treated in (A), and VEGF expression was analyzed by real-time PCR with GAPDH as the internal control. (E, F, G) Saos-2 cells were transiently transfected with an empty vector, hTERT-WT, and hTERT-DN. hTERT (E) and VEGF expression (F) were quantified by real-time PCR. Secreted VEGF was quantified by ELISA (G). Results were expressed as the mean ±SD from a minimum of three experiments.

First, we investigated the effect of telomerase inhibition on VEGF expression and secretion by three different gastrointestinal cell lines; AGS, Caco-2 and HepG2/C3A. Cells were treated for 48 h with telomerase inhibitors BIBR-1532 (10 μM) and costunolide (10 μM). We found that costunolide significantly decreased VEGF secretion in AGS (p = 0.0462), Caco2 (p<0.0001) and HepG2/C3A (p<0.0001), while BIBR significantly decreased VEGF secretion in HepG2/C3A cells (p = 0.0125) (Fig 1A). Accordingly, BIBR-1532 and costunolide significantly decreased VEGF mRNA levels in AGS (p<0.0001), Caco2 (p = 0.048) and HepG2/C3A (p = 0.0354) (Fig 1C).

The effect of telomerase inhibitors on VEGF secretion was confirmed by targeting hTERT with siRNA. hTERT knockdown significantly decreased VEGF secretion in AGS (P = 0.0163), Caco2 (p = 0.0046), and HepG2/C3A (p = 0.0014) (Fig 1B). Consequently, hTERT knockdown decreased VEGF expression 3-fold in AGS (p = 0.0131), 3.5-fold in Caco2 (p = 0.0179), and 2-fold in HepG2/C3A (p = 0.0162), 72 h post-transfection (Fig 1D). It is important to note that telomerase inhibitors BIBR-1532, costunolide and hTERT siRNA had no effect on VEGF secretion in Saos-2 (Fig 1A and 1B), which confirms that telomerase inhibition by these compounds is specific.

### hTERT expression in Saos-2 cells increases VEGF expression and secretion, independent of telomerase activity

The effect of telomerase on VEGF was studied in a telomerase negative, VEGF-secreting cell line, Saos-2. We transfected the cells with three different constructs: an empty vector as a control, a wild-type hTERT construct (hTERT-WT), and a mutated hTERT with no catalytic activity (hTERT-DN). hTERT-WT and hTERT-DN significantly increased hTERT expression 24 h post-transfection, and then hTERT expression decreased in both conditions after 48 h (Fig 1D). In accordance with the hTERT mRNA levels profile post-transfection, Saos-2 transfection with hTERT-WT and hTERT-DN increased VEGF mRNA levels 2-fold (p = 0.0031) and 1.5-fold (p = 0.0002), respectively (Fig 1E). Thus, hTERT-WT and hTERT-DN expression in Saos-2 increased VEGF secretion 44% (p = 0.042) and 31% (p = 0.0002), respectively (Fig 1F).

### Bevacizumab increases hTERT expression and telomerase activity

**Fig 2:**
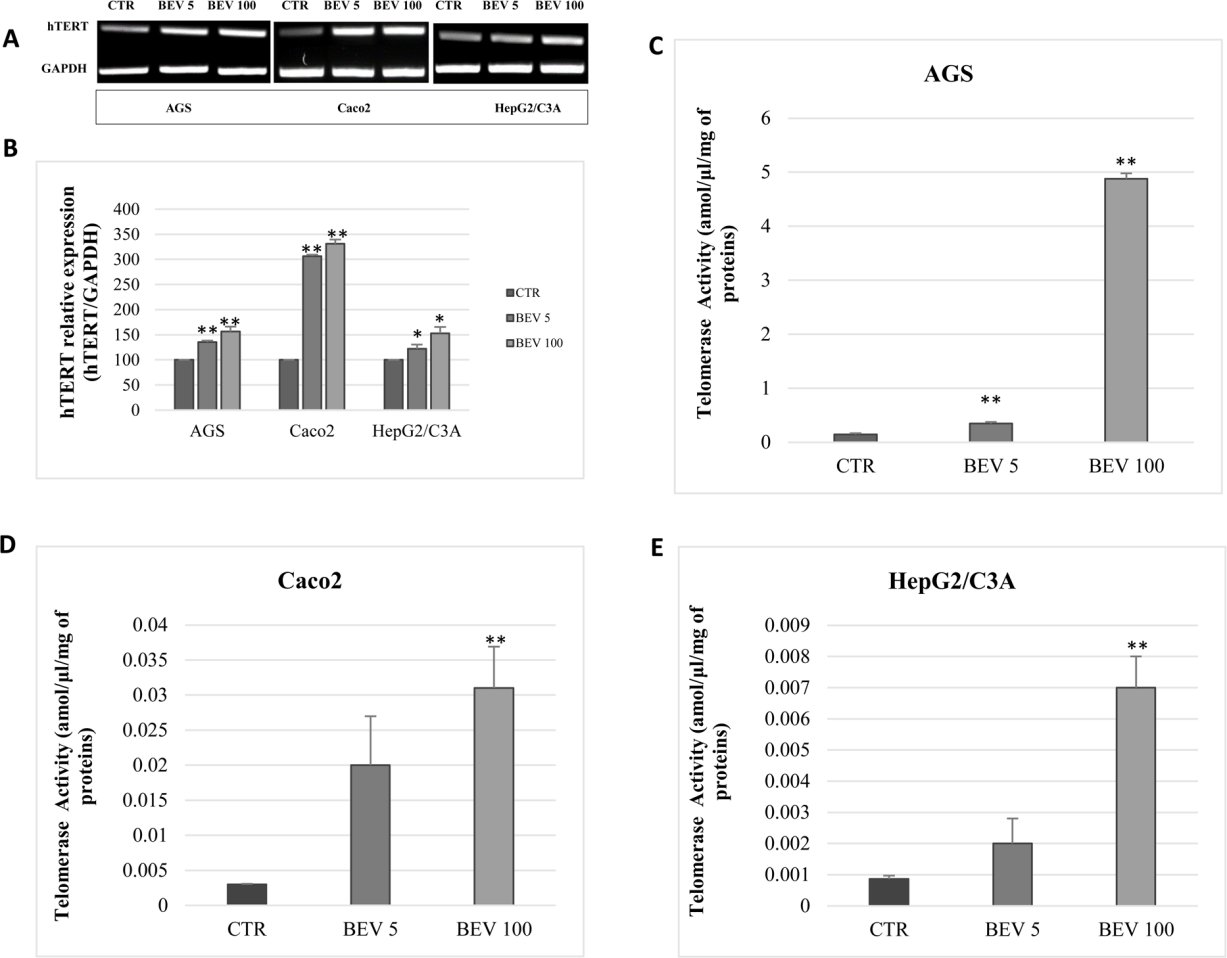
VEGF inhibition with bevacizumab increases hTERT expression and telomerase activity. (A) Semi-quantitative RT-PCR analysis of hTERT expression from AGS, Caco2, and HepG2/C3A cells treated for 48 h with bevacizumab at 5 ng/ml and 100 *μ*g/ml. GAPDH was used as a loading control. (B) The amount of transcripts referred to in (A) were quantified by real-time PCR with GAPDH as an internal control. (C, D, E) Telomerase activity was detected in AGS (C), Caco2 (D), and HepG2/C3A (E). Results were expressed as the mean ± SD from three experiments.

We next studied the effect of bevacizumab treatment on telomerase expression and activity. Interestingly, bevacizumab (5 ng/ml) increased hTERT mRNA levels 35.2% in AGS (P = 0.0002), 62.0% in Caco2 (p<0.0001), and 21.8% in HepG2/C3A (p = 0.03) (Fig 2A and 2B), while bevacizumab (100 μg/ml) increased hTERT mRNA levels 42.3% in AGS (p = 0.0046), 94.1% in Caco2 (p<0.0001), and 52.5% in HepG2/C3A (p = 0.0145) (Fig 2A and 2B). Accordingly, bevacizumab at 5 ng/ml and 100 μg/ml significantly increased hTERT mRNA levels (Fig 2A and 2B) and telomerase activity in AGS (Fig 2C), Caco2 (Fig 2D), and HepG2/C3A (Fig 2E).

### The effect of bevacizumab treatment on hTERT expression implicates PI3K, AKT, mTOR and HIF-1α

**Fig 3:**
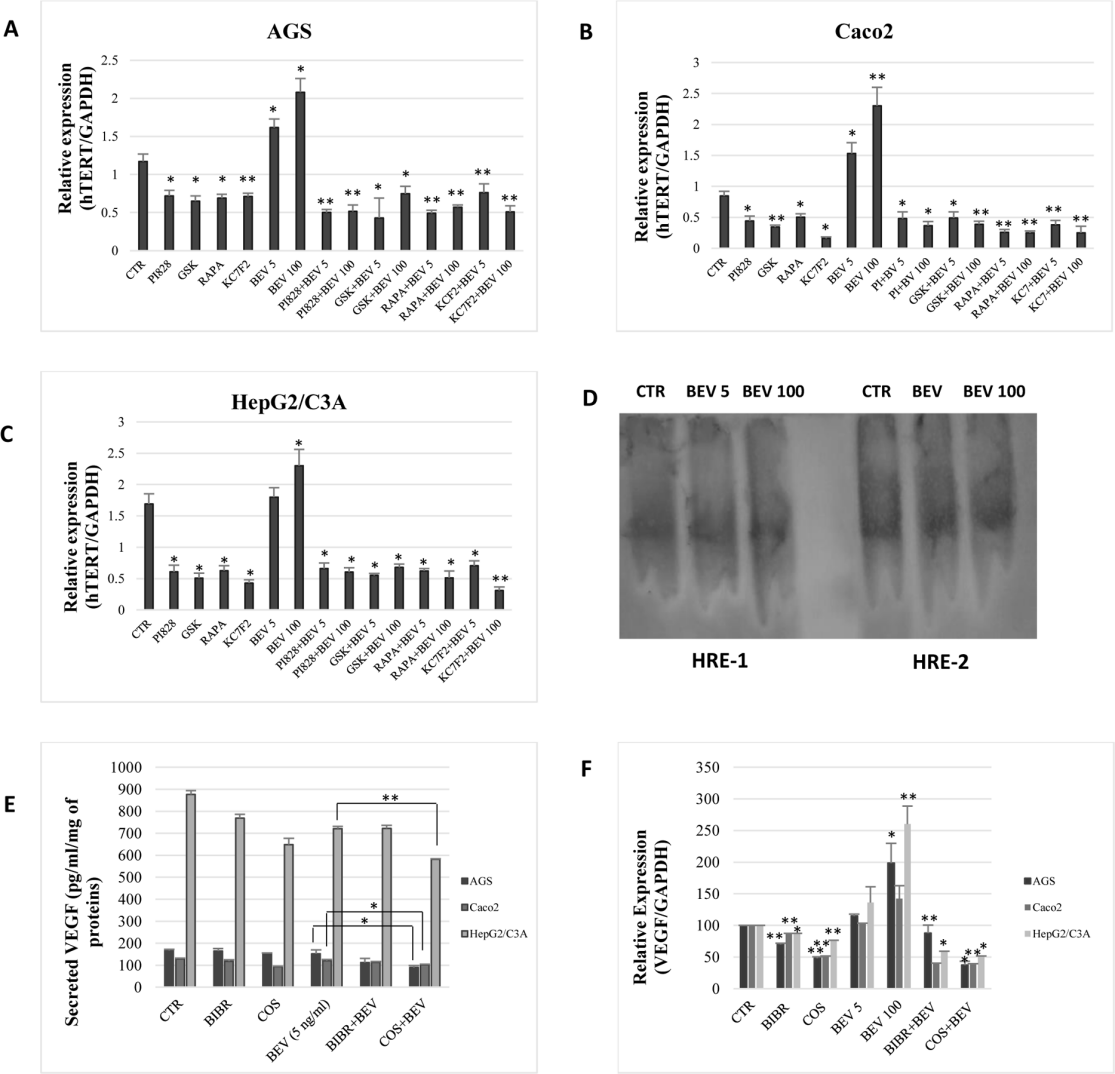
VEGF induces hTERT expression via PI3K/AKT/mTOR and HIF-1α. AGS (A), Caco2 (B), and HepG2/C3A (C) were treated with PI 828 (10*μ*M), GSK 690693 (100nM), Rapamycin (200nM), and KC7F2 (40*μ*M) with or without bevacizumab for 48 h. The amount of hTERT transcripts was quantified by real-time PCR. (D) EMSA was performed on nuclear cell extracts incubated with oligonucleotides of two HIF-1α binding sites, HRE-1 and HRE-2. (E) VEGF secretion was quantified with ELISA. (F) Total RNAs from the cells treated in (E) were collected, and the amount of hTERT transcripts was quantified by real-time PCR. Results were expressed as the mean ± SD from three experiments.

Because bevacizumab increased telomerase expression and activity in the studied cancer cell lines, we investigated whether the PI3K/AKT pathway could be implicated in this mechanism. Our results showed that the inhibition of PI3K, AKT, mTOR and HIF-1α using PI 828 (10μM), GSK-690693 (100 nM), Rapamycin (200 nM) and KC7F2 (40μM), respectively, significantly decreased hTERT mRNA levels in AGS, Caco2, and HepG2/C3A (Fig 3A, 3B and 3C). Interestingly, a combined inhibition of VEGF and the PI3K/AKT pathway or HIF-1α abrogates hTERT upregulation by bevacizumab (Fig 3A, 3B and 3C).

Moreover, we evaluated the effect of bevacizumab treatment on PI3K, AKT, and mTOR protein levels by flow cytometry. Interestingly, AGS, Caco2, and HepG2/C3A cells treatment with bevacizumab at 5 ng/ml and 100 μg/ml significantly increased PI3K, AKT, and mTOR expression (p<0.05) in the three cell lines (Table 1, S3–S5 Figs). These results indicate that bevacizumab treatment activates the PI3K/AKT pathway.

**Table 1:** The effect of bevacizumab treatment on PI3K, AKT, and mTOR expression.

		PI3K	AKT	mTOR
**AGS**	**CTR**	3.89±0.80	5.68±1.37	4.05±0.44
	**BEV 5**	6.31±1.89	6.22±1.40	6.9±0.20
	**BEV 100**	16.34±1.40	16.53±0.14	17.75±0.52
**Caco2**	**CTR**	5.91±0.64	17.24±0.22	15.10±0.12
	**BEV 5**	15.5±0.17	19.28±1.34	21.09±1.81
	**BEV 100**	21.19±1.87	19.3±2.13	28.04±0.80
**HepG2/C3A**	**CTR**	20.86±0.57	31.68±2.04	31.31±1.85
	**BEV 5**	38.01±0.60	38.97±0.71	36.84±3.49
	**BEV 100**	45.37±1.62	48.09±2.15	38.29±2.67

AGS, Caco2, and HepG2/C3A cells were treated with bevacizumab at 5 ng/ml and 100 μg/ml. After 48 hours, cells were collected, permeabilized and then stained with the appropriate monoclonal antibodies. Results were expressed as the mean ± SD from three experiments.

Furthermore, we investigated whether HIF-1α binds to hTERT promoter following cell treatment with bevacizumab. However, our results did not show a shift in DNA migration when the cells were treated with bevacizumab at 5 ng/ml and 100 μg/ml (Fig 3D).

### A combined inhibition of VEGF and telomerase reduces VEGF expression and secretion

**Fig 4:**
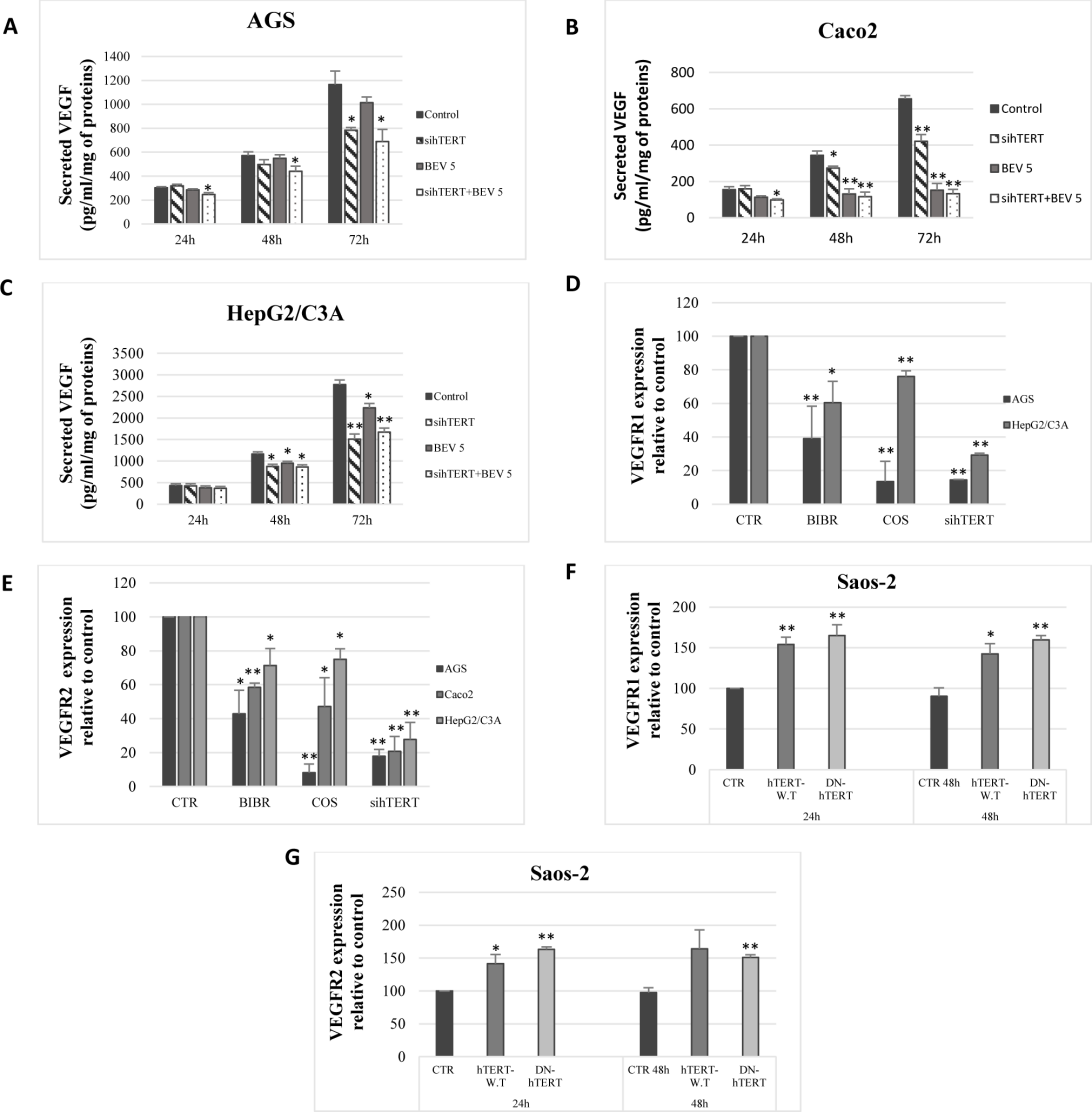
Telomerase regulates VEGF, VEGFR1, and VEGFR2 expression. AGS (A), Caco2 (B), and HepG2/C3A (C) cells were treated with control siRNA or hTERT siRNA with or without bevacizumab, and VEGF secretion was evaluated by ELISA. (D, E) Cells were treated with BIBR-1532, costunolide, or transiently transfected with hTERT siRNA for 72h. VEGFR1 (D) and VEGFR2 (E) transcript amounts were quantified with real-time PCR. (F, G) Saos-2 cells were transiently transfected with an empty vector, hTERT-WT or hTERT-DN. VEGFR1 (F) and VEGFR2 (G) were then quantified with real-time PCR. Results were expressed as the mean ± SD from three experiments.

As our results show, telomerase regulates VEGF expression while VEGF inhibition with bevacizumab increases telomerase expression and activity. Hence, we investigated whether a combined inhibition of VEGF and telomerase could inhibit this possible autocrine regulation and reduce the production of VEGF. Interestingly, our results show that the combination of bevacizumab (5 ng/ml) to costunolide (Fig 3E and 3F) and hTERT siRNA (Fig 4A, 4B and 4C) significantly reduced VEGF expression and secretion in AGS, Caco2, and HepG2/C3A. These results indicate that the combination of a relatively low concentration of bevacizumab with telomerase inhibitors enhances VEGF inhibition on protein and mRNA levels.

### hTERT regulates VEGFR1 and VEGFR2 expression in AGS, Caco2, HepG2/C3A, and Saos-2

For a better understanding of the connection between VEGF and telomerase, we investigated whether the VEGF receptors are implicated in this mechanism. Using HUVECs as a positive control, our results showed that AGS, Caco2, HepG2/C3A, and Saos-2 expressed, at the mRNA level, VEGFR1 and VEGFR2, whereas Caco2 only expressed VEGFR2. Interestingly, telomerase inhibition with BIBR-1532, costunolide, and hTERT knockdown significantly decreased VEGFR1 (Fig 4D) and VEGFR2 (Fig 4E) expression in AGS, Caco2, and HepG2/C3A. Moreover, hTERT overexpression in Saos-2 with hTERT-WT and hTERT-DN constructs, significantly increased VEGFR1 (Fig 4F) and VEGFR2 (Fig 4G) expression levels 24h post-transfection. These results indicate that telomerase regulates VEGFR1 and VEGFR2 expression in cancer cells through its catalytic subunit hTERT.

### Bevacizumab increases VEGFR1 and VEGFR2 expression in AGS, Caco2, and HepG2/C3A through hTERT

**Fig 5:**
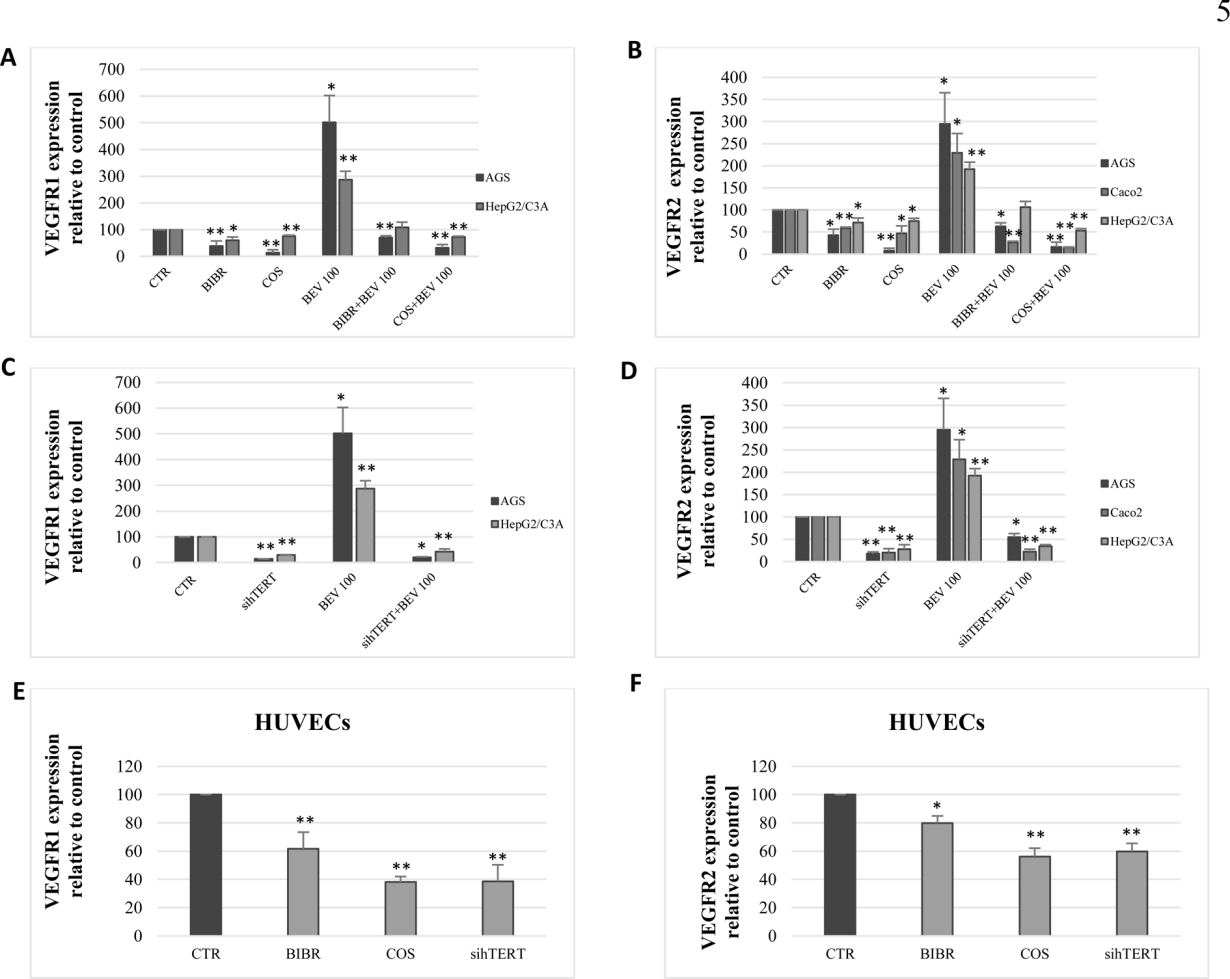
Bevacizumab increases VEGFR1 and VEGFR2 expression. (A, B) Telomerase inhibitors were combined to bevacizumab (100 *μ*g/ml) and then VEGFR1 (A) and VEGFR2 (B) were quantified by real-time PCR. (C, D) Cells were transiently transfected with either control siRNA or hTERT siRNA and treated with bevacizumab (100 *μ*g/ml). VEGFR1 (C) and VEGFR2 (D) were quantified by real-time qPCR. (E, F) HUVECs were cultured in the presence of telomerase inhibitors for 48 h and hTERT siRNA for 72 h. The expression of VEGFR1 (E) and VEGFR2 (F) were quantified by real-time PCR. Results were expressed as the mean ± SD from three experiments.

Given that bevacizumab increases hTERT expression, while hTERT regulates VEGFR1 and VEGFR2 expression, we investigated whether bevacizumab treatment increases VEGFR1 and VEGFR2 mRNA levels. Cell treatment with bevacizumab significantly increased VEGFR1 (Fig 5A) and VEGFR2 (Fig 5B) expression in AGS, Caco2, and HepG2/C3A. Interestingly, combining telomerase inhibitors to bevacizumab inhibits VEGFR1 (Fig 5A and 5C) and VEGFR2 (Fig 5B and 5D) upregulation caused by bevacizumab alone.

Because VEGFR2 is cited as the VEGF receptor with the most important kinase activity [37,38], we evaluated VEGFR2 protein expression in AGS, Caco2, and HepG2/C3A. However, we did not detect the expression of VEGFR2 at the protein level in these cells.

### hTERT regulates VEGFR1 and VEGFR2 expression in HUVECs

**Fig 6:**
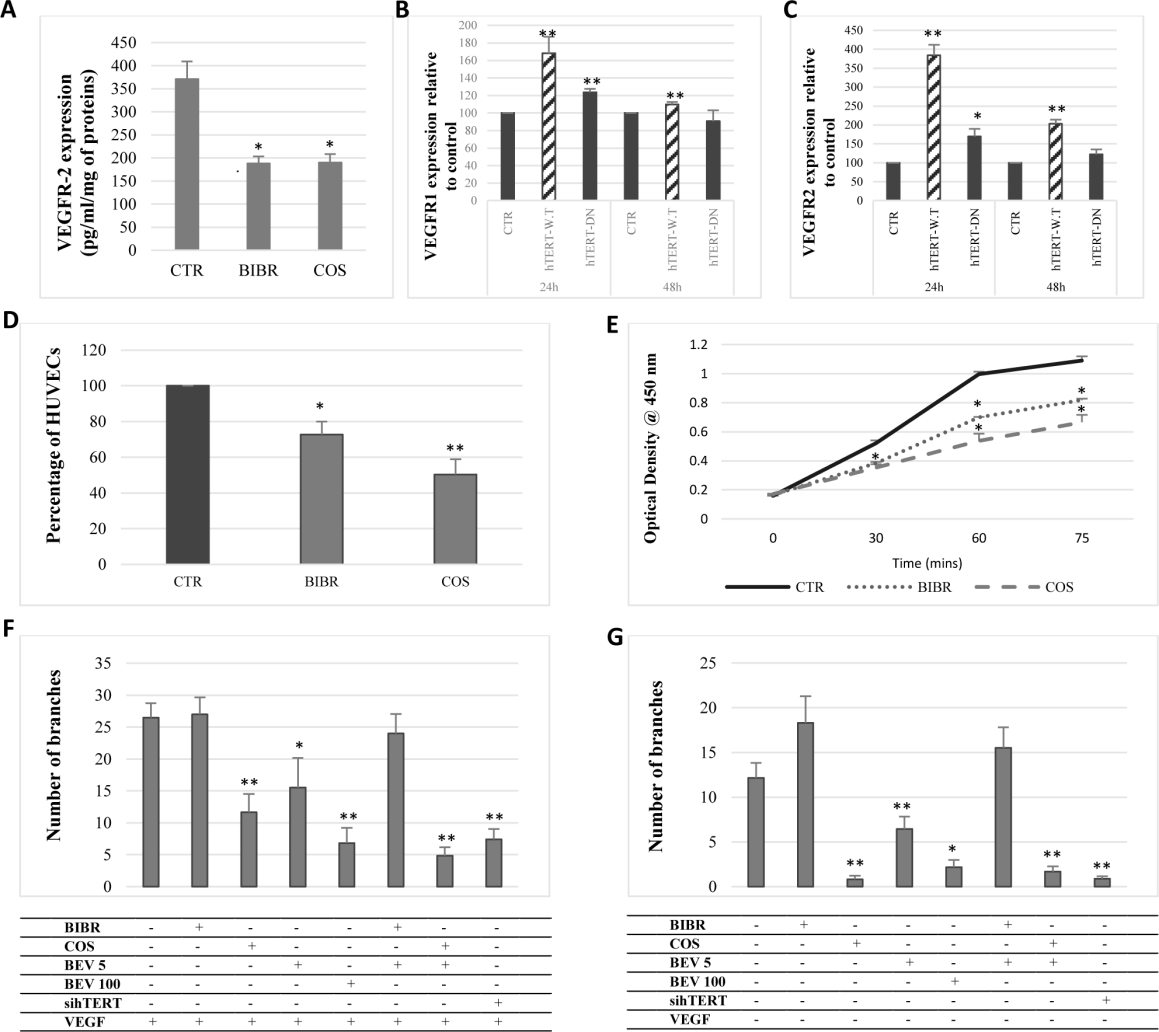
Telomerase inhibition decreases the proliferation and tube formation of HUVECs. (A) HUVECs were treated with BIBR-1532 and costunolide. VEGFR2 protein levels were quantified by ELISA using the cell protein extracts. (B, C) HUVECs were transiently transfected with either a control siRNA or hTERT siRNA for 72 h. VEGFR1 and VEGFR2 expression was then assessed by real-time PCR. (D) HUVECs treated with BIBR-1532 and costunolide were collected and counted using trypan blue. (E) HUVECs were cultured in a 96-well plate and treated with telomerase inhibitors. Tetrazolium salt was added to each well and changes in absorbance following formazan formation was detected at 450 nm. (F, G) HUVECs were cultured on an ECM in the presence of recombinant VEGF_165_ (50 ng/ml), telomerase inhibitors and bevacizumab. Capillary-like structures were quantified in the presence (F) and absence (G) of recombinant VEGF_165_.Values are represented as the mean ± SE of 10 randomly chosen fields.

According to our results, telomerase regulates VEGFR1 and VEGFR2 expression in gastrointestinal cancer cells. Hence, we evaluated VEGFRs’ response to telomerase inhibition and overexpression in HUVECs. Our results showed that telomerase inhibition with BIBR-1532 and costunolide and hTERT siRNA significantly decreased VEGFR1 and VEGFR2 expression (p<0.05) (Fig 5E and 5F) and protein levels (p = 0.01) (Fig 6A). Moreover, hTERT overexpression in HUVECs significantly increased VEGFR1 (p<0.01) (Fig 6B) and VEGFR2 (p<0.005) (Fig 6C) mRNA levels 24h post-transfection with hTERT-WT and hTERT-DN.

### Telomerase inhibition reduces the proliferation and tube formation by HUVECs

**Fig 7:**
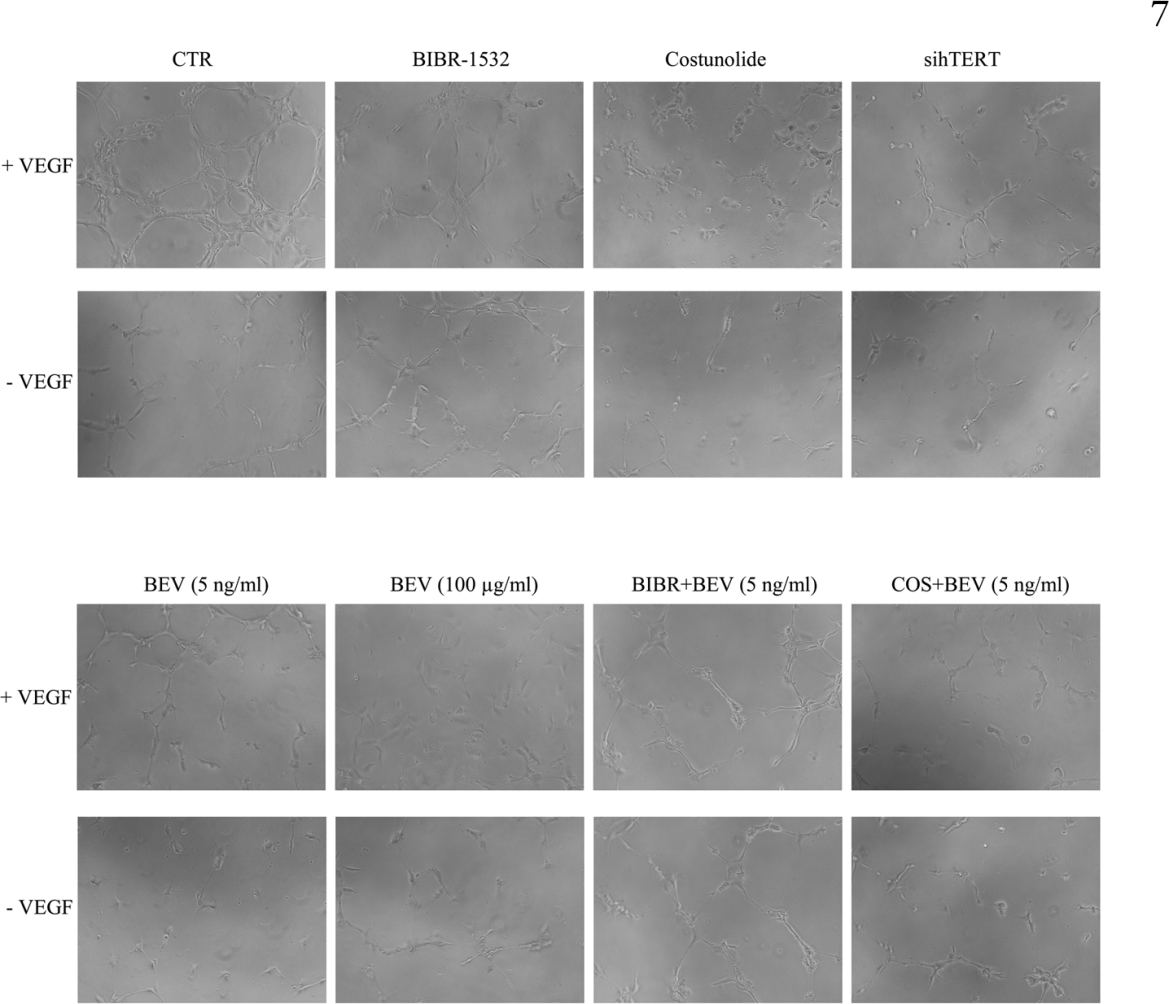
Telomerase regulates tube formation by HUVECs. HUVECs previously treated with BIBR-1532 (10 μM), costunolide (10 μM), bevacizumab (5 ng/ml or 100 μg/ml), or hTERT siRNA were cultured on an extracellular matrix (ECM) in the presence or absence of recombinant VEGF165 (50 ng/ml). Angiogenesis began after 4 hours, and tube formation was then visualized after 6 hours, using an inverted microscope (100X).

Because telomerase regulates the expression of VEGFR1 and VEGFR2 in HUVECs, we investigated the effect of telomerase inhibition on the proliferation of HUVECs and their tube formation capability. Indeed, our results showed that BIBR-1532 and costunolide significantly reduce HUVECs’ proliferation (p = 0.01) (Fig 6D) and cell number (p<0.01) (Fig 6E). Furthermore, HUVECs treated with telomerase inhibitors and bevacizumab were cultured on an ECM, and tube formation was then evaluated after 6h, in the presence and in the absence of recombinant VEGF (50 ng/ml). Interestingly, costunolide and hTERT knockdown significantly decreased the number of branches in the presence or absence of VEGF (Fig 6F and 6G and Fig 7), and the combination of bevacizumab to costunolide further reduced the number of branches (Fig 7). These results show that telomerase inhibition reduces the tube formation capability of endothelial cells, even in the presence of VEGF, which indicates the importance of telomerase in angiogenesis.

## Discussion

The VEGF and VEGFR signaling pathways were the prime targets for developing anti-angiogenic cancer therapy. However, a number of patients treated with various anti-VEGF agents such as bevacizumab, did not obtain a pronounced benefit, which was most likely due to resistance mechanisms [26]. Telomerase is a ribonucleoprotein expressed in 85–95% of human cancers [4,5] and was shown to immortalize cancer cells and regulate VEGF expression and secretion. The ultimate aim of our study was to investigate a possible relationship between telomerase and VEGF in gastrointestinal cancers.

We first studied the effect of telomerase inhibition or overexpression on VEGF expression and secretion. In agreement with other studies [21,29,30], we found that telomerase inhibition decreased VEGF expression and secretion in AGS, Caco2, and HepG2/C3A, while hTERT overexpression in telomerase-negative cells, Saos-2, increased VEGF expression and secretion independently of telomerase activity. Hence, our results confirm that telomerase regulates VEGF expression and secretion in cancer cells, one of the other extra-telomeric roles of telomerase not related to its activity.

While most of the research conducted focused on VEGF regulation by telomerase, a limited number of studies examined the effect of VEGF inhibition on telomerase expression and activity in cancer cells. In this study, we partially inhibited the VEGF effect by bevacizumab. Moreover, we found that cell treatment with bevacizumab increased hTERT expression and telomerase activity in AGS, Caco2, and HepG2/C3A, 48h post-treatment.

Indeed, a number of studies reported that the inhibition of VEGF signaling in cancer is related to a number of toxicities, resistance, and an increased invasiveness [24,39,40]. According to our results, VEGF inhibition also induces hTERT expression and telomerase activity. This effect could be related to the implication of telomerase in several extra-telomeric cellular functions, including gene expression, cellular signaling, and cell cycle [6–9]. Altogether, our results suggest that telomerase could be involved in the resistance mechanisms triggered by bevacizumab treatment.

VEGF inhibition with bevacizumab could modulate a signaling cascade that enhances hTERT transcription. Zaccagnini et al. reported that VEGF induces hTERT expression and telomerase activity via the PI3K/AKT pathway [29]. This prompted us to investigate whether the PI3K-AKT pathway is involved in telomerase activation by VEGF. Intriguingly, we found that bevacizumab treatment increases PI3K, AKT, and mTOR expression and that the inhibition of this pathway abrogates hTERT transcription upregulation by bevacizumab. Further, a number of studies suggested the presence of putative hypoxia responsive elements (HRE) in hTERT promoter and showed that HIF-1α upregulates hTERT directly by binding to these sites [41,42]. Hence, we investigated whether HIF-1α is implicated in hTERT transcription activation by VEGF. Our results showed that HIF-1α is implicated in hTERT regulation by VEGF because its inhibition abrogates the autoregulatory mechanism conducted by VEGF. However, the electrophoretic mobility shift assay did not show a binding of HIF-1α to hTERT promoter at two different HREs following VEGF inhibition with bevacizumab. Our results are not confirmative because it appears that HIF-1α could bind to other sites inside hTERT promoter. According to a study by Anderson et al., HIF-1α binds to HREs and to other downstream intragenic sites in hTERT promoter [43]. It is important to note that hypoxia, through HIF-1α, is considered an important mediator of angiogenesis and a possible contributor in the emergent resistance to angiogenesis inhibitors [44,45]

Our results suggest that VEGF inhibition upregulates hTERT transcription in an autoregulatory mechanism in order to enhance VEGF production by cancer cells (S2 Fig). Hence, we investigated whether a combined inhibition of VEGF and hTERT could abrogate this autoregulatory mechanism and reduce VEGF production. Therefore, we combined bevacizumab to telomerase inhibitors and hTERT siRNA. Interestingly, we found that a combined inhibition of telomerase and VEGF further reduces VEGF expression and secretion. However, a combined inhibition of telomerase and VEGF did not completely abrogate VEGF secretion by cancer cells, which indicates that VEGF secretion could be managed by other signaling pathways.

To identify the extracellular components involved in VEGF autocrine regulation, we investigated the presence and the modulation of VEGFR1 and VEGFR2 expression in the studied cancer cells, as the presence of these two receptors was reported in a number of cancer cell lines [31,32,34]. We noticed that AGS, HepG2/C3A, and Saos-2 expressed VEGFR1 and VEGFR2 at the mRNA level, while Caco2 only expressed VEGFR2. Remarkably, the inhibition of telomerase decreased VEGFR1 and VEGFR2 mRNA levels, while hTERT overexpression in Saos-2 increased VEGFR1 and VEGFR2 mRNA levels independently of telomerase activity. Considering its importance in VEGF-mediated signaling [46,47], we searched for the protein expression of VEGFR2. However, we did not detect VEGFR2 protein expression on the surface of the studied cancer cells. These results could indicate that the autocrine regulatory effects of VEGF could be conducted through VEGFR1 or other VEGF receptors.

Furthermore, our results show that bevacizumab treatment increases VEGF receptor expression in HUVECs through telomerase. Indeed, hTERT regulates VEGFR1 and VEGFR2 expression in HUVECs, and a combined inhibition of telomerase and VEGF abrogates VEGF receptor upregulation by VEGF. While VEGFR2 is known for its important role in inducing angiogenic signaling, VEGFR1 was reported as a decoy receptor and an inhibitor of VEGF signaling [48]. However, VEGFR1 inhibition was shown to reduce tumor growth in vivo and in vitro [49,50], which indicates that VEGFR1 could also contribute to an increased angiogenesis. Thus, inhibiting telomerase in endothelial cells could reduce angiogenesis by reducing VEGFR1 and VEGFR2 receptor expression, which nominates telomerase as a possible target for the regulation of tumor angiogenesis.

The effect of telomerase on VEGF receptor expression was translated by the change of HUVECs’ ability to form tubes when placed on an extracellular matrix. While telomerase inhibitors decreased HUVECs’ proliferation rate, tube-formation ability was only affected when hTERT was targeted with siRNA and costunolide. These results show that the angiogenic process of HUVECs depends on hTERT protein expression, rather than telomerase telomere-elongation activity. Interestingly, tube formation was inhibited by costunolide and hTERT knockdown in the presence and in the absence of VEGF, which indicates that telomerase inhibition could abrogate the effects of the angiogenic stimulation of HUVECs by VEGF. It is also important to note that a combined inhibition of telomerase and VEGF with bevacizumab reduces tube formation by HUVECs. Hence, in addition to the regulation of VEGFR1 and VEGFR2 expression in HUVECs, telomerase could be considered as an important therapeutic target for angiogenesis, considering its effects on HUVECs’ proliferation and tube-formation capacity, even in the presence of VEGF.

In conclusion, here we document for the first time the effect of the VEGF monoclonal antibody, bevacizumab, on telomerase expression and activity in gastrointestinal cancer cells. In this report, we suggest that telomerase could be implicated in the reduced survival benefit and the increased tumor resistance and invasion caused by the inhibition of VEGF signaling in patients with gastrointestinal cancers. Our results highlight the importance of telomerase in the regulation of angiogenesis and suggest that the inhibition of VEGF in combination with telomerase, PI3K/AKT, or HIF-1α inhibitors could improve tumor cell response to VEGF inhibitors and could decrease resistance mechanisms related to anti-VEGF treatments. However, the *in vitro* observations should be confirmed by *in vivo* experiments and be extended further by trying multiple drug combinations as a potential therapeutic strategy.

## Supporting information

S1 Fig Flow cytometry histograms of the HUVECs. The percentage of cells that stained positive is indicated in the upper right corner of each panel.
(TIF)

S2 Fig Proposed cell response to VEGF inhibition with bevacizumab. Cancer cells secrete VEGF that stimulates both endothelial cells and cancer cells via a paracrine and autocrine mechanism, respectively. (A) Following its secretion, VEGF would supposedly bind to VEGF receptors expressed by cancer cells. VEGF binding to the receptor would trigger receptor kinase domain phosphorylation and activate two different signaling cascades: an activating and an inhibiting pathway. The activating pathway involves PI3K, AKT, mTOR, and HIF-1α. HIF-1α would then bind to hypoxia responsive elements in hTERT promoter and activate its transcription. The other signaling pathway would activate c-Myc binding to the E-box in hTERT promoter and inhibit hTERT transcription [29]. The increase in hTERT expression induces VEGF secretion and VEGF receptors expression. (B) Following bevacizumab treatment, antibodies inhibit VEGF binding to the VEGF receptors. In an autocrine feedback regulation mechanism, the VEGF receptor would enhance PI3K/AKT pathway activation and upregulate hTERT transcription and protein levels in order to increase VEGF secretion and VEGF receptor expression.
(TIF)

S3 Fig Flow cytometry analysis of PI3K, AKT, and mTOR expression in AGS cells. AGS cells were treated with bevacizumab at 5 ng/ml and 100 μg/ml for 48 hours then cells were collected, permeabilized and stained with monoclonal antibodies against PI3K (A), AKT (B), and mTOR (C). The corresponding results are illustrated in the table (D) and expressed as the mean ± SD from three experiments.
(TIF)

S4 Fig Flow cytometry analysis of PI3K, AKT, and mTOR expression in Caco-2 cells. Caco-2 cells were treated with bevacizumab at 5 ng/ml and 100 μg/ml for 48 hours then cells were collected, permeabilized and stained with monoclonal antibodies against PI3K (A), AKT (B), and mTOR (C). The corresponding results are illustrated in the table (D) and expressed as the mean ± SD from three experiments.
(TIF)

S5 Fig Flow cytometry analysis of PI3K, AKT, and mTOR expression in HepG2/C3A cells. HepG2/C3A cells were treated with bevacizumab at 5 ng/ml and 100 μg/ml for 48 hours then cells were collected, permeabilized and stained with monoclonal antibodies against PI3K (A), AKT (B), and mTOR (C). The corresponding results are illustrated in the table (D) and expressed as the mean ± SD from three experiments.
(TIF)
