# Supplementary figures and images for: Gastrointestinal cancer cells treatment with bevacizumab activates a VEGF autoregulatory mechanism involving telomerase catalytic subunit hTERT via PI3K-AKT, HIF-1α and VEGF receptors
Source: PLoS One. 2017 Jun 8;12(6):e0179202. doi: 10.1371/journal.pone.0179202 (PMC5466359; doi:10.1371/journal.pone.0179202)

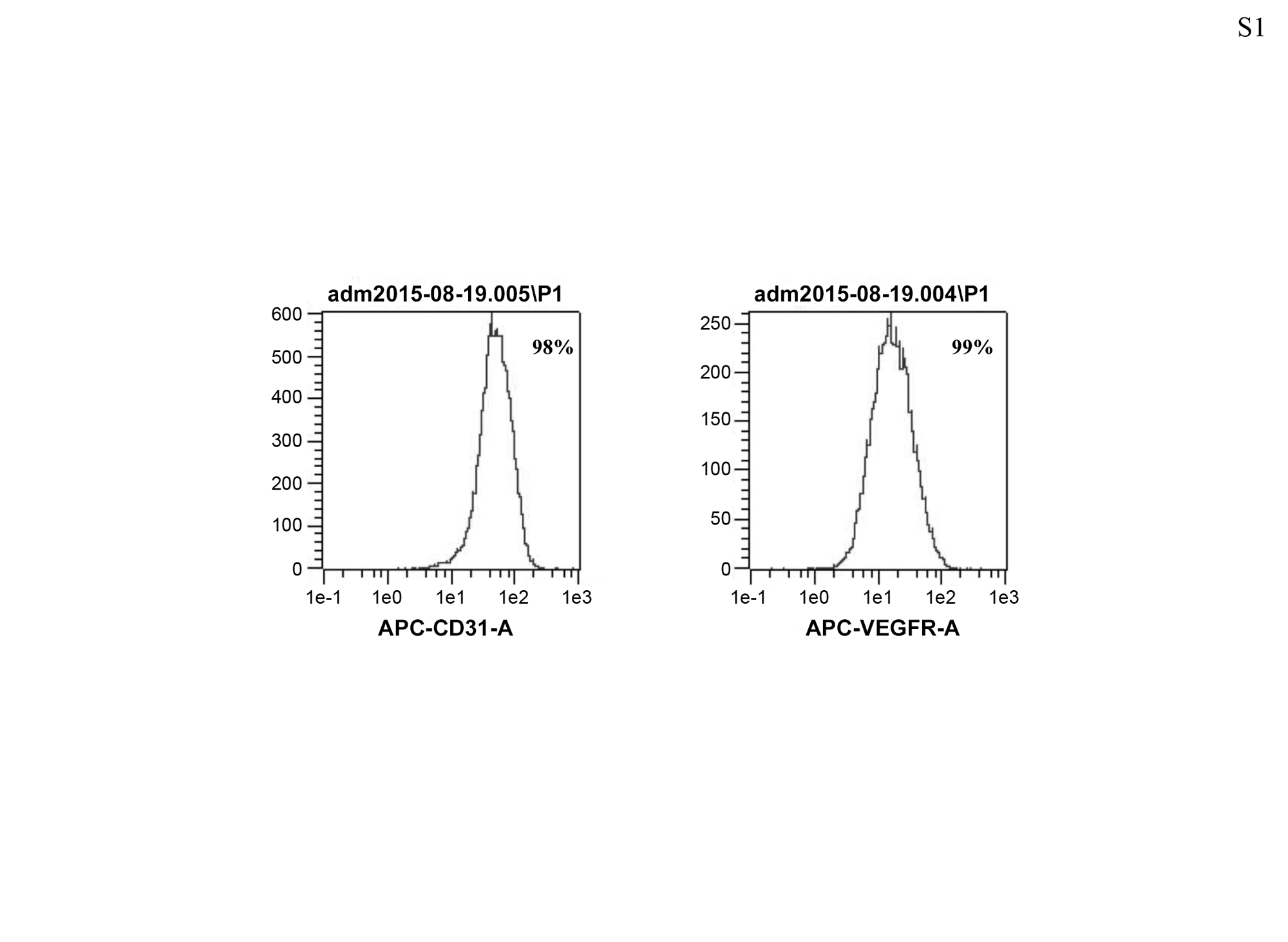

Supplement: S1 Fig — The percentage of cells that stained positive is indicated in the upper right corner of each panel. (TIF) [file pone.0179202.s001.tif]

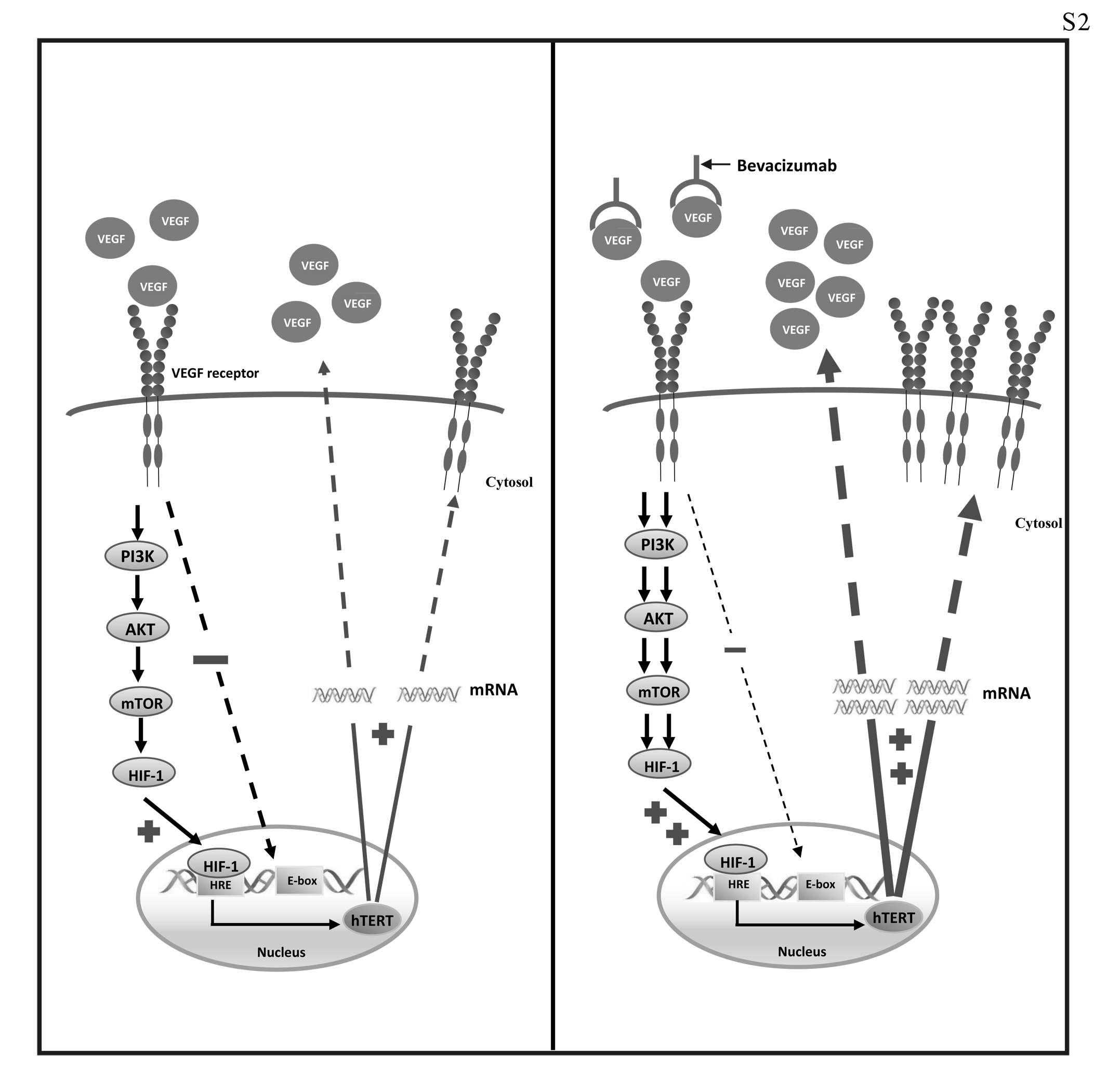

Supplement: S2 Fig — Cancer cells secrete VEGF that stimulates both endothelial cells and cancer cells via a paracrine and autocrine mechanism, respectively. (A) Following its secretion, VEGF would supposedly bind to VEGF receptors expressed by cancer cells. VEGF binding to the receptor would trigger receptor kinase domain phosphorylation and activate two different signaling cascades: an activating and an inhibiting pathway. The activating pathway involves PI3K, AKT, mTOR, and HIF-1α. HIF-1α would then bind to hypoxia responsive elements in hTERT promoter and activate its transcription. The other signaling pathway would activate c-Myc binding to the E-box in hTERT promoter and inhibit hTERT transcription [29]. The increase in hTERT expression induces VEGF secretion and VEGF receptors expression. (B) Following bevacizumab treatment, antibodies inhibit VEGF binding to the VEGF receptors. In an autocrine feedback regulation mechanism, the VEGF receptor would enhance PI3K/AKT pathway activation and upregulate hTERT transcription and protein levels in order to increase VEGF secretion and VEGF receptor expression. (TIF) [file pone.0179202.s002.tif]
